# The Other Side of Plastics: Bioplastic-Based Nanoparticles for Drug Delivery Systems in the Brain

**DOI:** 10.3390/pharmaceutics15112549

**Published:** 2023-10-28

**Authors:** Erwin Pavel Lamparelli, Marianna Marino, Marta Anna Szychlinska, Natalia Della Rocca, Maria Camilla Ciardulli, Pasqualina Scala, Raffaella D’Auria, Antonino Testa, Andrea Viggiano, Francesco Cappello, Rosaria Meccariello, Giovanna Della Porta, Antonietta Santoro

**Affiliations:** 1 Department of Medicine, Surgery and Dentistry, University of Salerno, 84081 Baronissi, Italy; elamparelli@unisa.it (E.P.L.); mamarino@unisa.it (M.M.); nataliadellarocca95@gmail.com (N.D.R.); mciardulli@unisa.it (M.C.C.); pscala@unisa.it (P.S.); radauria@unisa.it (R.D.); aviggiano@unisa.it (A.V.); ansantoro@unisa.it (A.S.); 2 Faculty of Medicine and Surgery, Kore University of Enna, Cittadella Universitaria, 94100 Enna, Italy; martaanna.szychlinska@unikore.it; 3 Department of Environmental, Biological and Pharmaceutical Sciences and Technologies, University of Campania “Luigi Vanvitelli”, 81100 Caserta, Italy; antonino.testa@unicampania.it; 4 Department of Biomedicine, Neuroscience and Advanced Diagnostics, University of Palermo, 90127 Palermo, Italy; francesco.cappello@unipa.it; 5 Euro-Mediterranean Institute of Science and Technology (IEMEST), 90139 Palermo, Italy; 6 Department of Movement and Wellbeing Sciences, Parthenope University of Naples, 80133 Naples, Italy; rosaria.meccariello@uniparthenope.it; 7 Research Centre for Biomaterials BIONAM, University of Salerno, Via Giovanni Paolo II, 84084 Fisciano, Italy

**Keywords:** bioplastics, nanoparticles, drug delivery systems, brain

## Abstract

Plastics have changed human lives, finding a broad range of applications from packaging to medical devices. However, plastics can degrade into microscopic forms known as micro- and nanoplastics, which have raised concerns about their accumulation in the environment but mainly about the potential risk to human health. Recently, biodegradable plastic materials have been introduced on the market. These polymers are biodegradable but also bioresorbable and, indeed, are fundamental tools for drug formulations, thanks to their transient ability to pass through biological barriers and concentrate in specific tissues. However, this “other side” of bioplastics raises concerns about their toxic potential, in the form of micro- and nanoparticles, due to easier and faster tissue accumulation, with unknown long-term biological effects. This review aims to provide an update on bioplastic-based particles by analyzing the advantages and drawbacks of their potential use as components of innovative formulations for brain diseases. However, a critical analysis of the literature indicates the need for further studies to assess the safety of bioplastic micro- and nanoparticles despite they appear as promising tools for several nanomedicine applications.

## 1. Introduction

Plastic diffusion is deemed a significant indicator of the onset of the Anthropocene, [1] an era in which humans altered and dominated the Earth and its ecosystems [2]. Despite the convenience aspects, the widespread use of plastics and their uncontrolled waste has resulted in negative impacts on the environment and human health [3]. In addition, most plastic products are added with various chemical compounds to improve functional properties, such as plasticizers (phthalates), flame retardants, antioxidants (i.e., IRGAFOS-168), acid scavengers, stabilizers (e.g., bisphenol A, BPA), and pigments [4]. Plastic degradation produces microplastics (MPs, particle size lower than 5 mm) and nanoplastics (NPs, size less than 1 µm) [5] that cross biological barriers and accumulate in the food chain [6].

More recently, “environmentally sustainable” plastic materials fabricated adopting biodegradable polymers, such as polylactic acid (PLA) and poly-lactic-co-glycolic acid (PLGA), have been introduced on the market. These bioplastics undergo a more rapid degradation in the environment; indeed, these biopolymers have been developed for pharmaceutical and biomedical applications in order to have a transient polymer (bioresorbable) but with mechanical properties similar to the non-biodegradable ones [7]. However, the recent widespread use of bioplastic-based materials needs a critical approach because the polymer’s faster biodegradation poses an issue for a more rapid accumulation in the form of particles in living tissues.

This issue is confirmed by the fact that these polymers are properly adopted in the form of micro- and nanoparticles as drug carriers in pharmaceutical formulations. For example, bioplastic-based NPs can be targeted and accumulated (depot systems) in a given tissue in order to achieve a proper sustained release of the loaded drug [8,9]. Bioplastic NPs can also prolong the therapeutic effect of a given drug, improving its efficacy [9]. Recent studies also suggest that specific concentrations of bioplastic NPs in the central nervous system (CNS) promote their passage through the blood–brain barrier (BBB) or blood–cerebrospinal fluid barrier (BCSFB). Indeed, bioplastic NPs seemed able to cross the BBB through transcytosis pathways and proper surface modifications can allow their passage through the BBB via receptor-mediated endocytosis or to deeply diffuse in the brain parenchyma [10]. This behavior, while extremely interesting for the development of new drug formulations for the CNS, poses significant challenges in terms of cost, failure, and clinical implementation; on the other hand, it may also raise public health concerns. 

Hence, the aim of this review is to provide a different point of view on bioplastics and their degradation products. Truly, bioplastic MPs and NPs should be considered particularly effective for pharmaceutical formulations and precision medicine, to transport drugs into organs, like the brain, that are protected by biological barriers. Indeed, bioplastic MPs/NPs will be discussed as successful tools for brain drug delivery; however, different particle accumulation of bioplastics in the environment may enhance the issues for potential tissue accumulation, with unknown long-term biological effects. 

## 2. Bioplastics: Definition, Chemical Properties, and Applications

Bioplastics can be divided into two categories: biodegradable and biobased [11,12,13]. Biobased plastics are entirely or partially made from biological resources and are not necessarily biodegradable. Plastics’ biodegradability is determined by the chemical composition of the polymer and environmental conditions [14]. On the other hand, biotic degradation is a process in which microorganisms, such as fungi or bacteria, reduce polymeric structure into smaller molecules that are utilized as a source of carbon or energy [15]. Photodegradation and hydrolysis consist of chemical processes in which high-energy radiations (UV) and water molecules induce polymer chain degradation [16,17,18]. In polymer degradation, biotic and abiotic factors can sometimes act together. Typically, abiotic degradation produces small fragments of plastic, which are subsequently degraded by microorganisms [18]. However, this process inevitably leads to the formation of small plastic particles with different characteristics and size [11,19]. 

**Figure 1:**
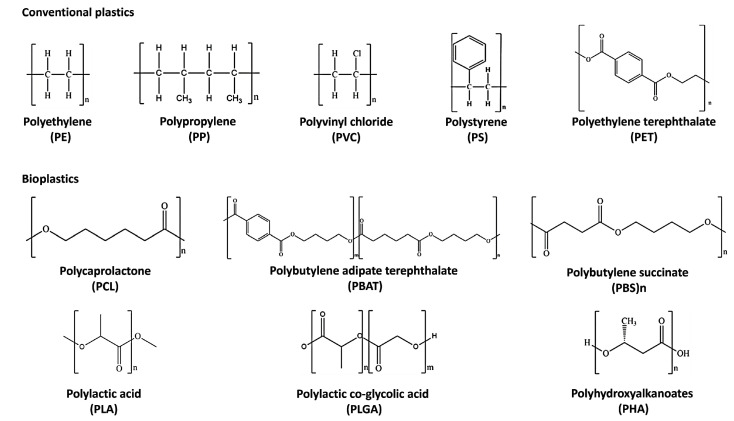
Chemical structures of the most diffused conventional plastics and bioplastics.

Non-biodegradable plastics or petroleum-based plastics (conventional plastics) include polyethylene (PE), polypropylene (PP), polyvinyl chloride (PVC), and polystyrene (PS), which belong to the polyolefin class [20]. They are thermoplastic polymers in which olefin monomer units like ethylene, styrene, and vinyl chloride are combined to form long chains [21,22] (Figure 1). Polyolefins represent the leading industrial polymers due to their remarkable chemical stability and mechanical characteristics [23]. The manufacturing processes of these plastics and applications have been well described elsewhere [24,25,26,27,28,29,30,31,32,33,34]. The so-called “biobased” plastics, such as bio-PE obtained from sugar cane [35] and bio-PET produced by the oxidization of bio-ethylene derived from the fermentation of glucose [36], find many applications in the packaging sector, particularly for drinking bottles and textile industries; however, they are non-biodegradable [37].

Biodegradable plastics comprise poly-caprolactone (PCL), poly-butylene succinate (PBS), poly-butylene adipate terephthalate (PBAT), poly-lactic acid (PLA), poly-lactic-co-glycolic acid (PLGA), and poly-hydroxy-alkanoate (PHA) [14] (Figure 1). PCL is suitable for a wide range of medical applications such as implantable biomedical devices, sutures, and tissue engineering scaffolds due to its biocompatibility and slow degradation [38,39,40]. It is used for the synthesis of green polyurethane [41] and commonly blended with biobased biodegradable plastics [42] to improve its thermal and mechanical properties [43]. On the contrary, PBS has a relatively slow biodegradation rate and biocompatibility [43].

PLA is made from 100% bioresources and is totally biodegradable and recyclable [44]. It is produced by a combination of lactic acid monomers derived from the fermentation of sugars obtained by sugar cane, potatoes, and corn [45,46]. PLA degrades in 6 to 24 months in the environment, depending on various factors, such as temperature, product size and shape, and isomer ratio. Despite some inherent weaknesses like brittleness and moisture uptake, it exhibits good thermomechanical properties like the traditional plastics PET and PP and has been extensively applied in different fields, ranging from packaging applications, bowls, films, and bottles, to clothes, textile furniture, hygiene products, and mulch films for agriculture [47,48,49]. Furthermore, it can be also copolymerized with polyethylene glycol (PEG) to enhance its hydrophilic and biocompatibility properties, making it suitable for drug delivery systems [50]. However, despite its eco-friendly characteristics, the commercial production of PLA is hindered by the high cost of raw materials and the lack of composting infrastructure in most markets. Implementing composting infrastructure would enable the widespread use of PLA and would reduce the environmental impact of traditional plastics [51]. 

PLGA is another bioplastic component frequently used as a copolymer of polyglycolic acid (PGA) and PLA. In fact, it is frequently employed in biomedical applications, due to its biocompatibility and fast biodegradation. PLGA, like PLA, can be produced by polycondensation or ring-opening polymerization, varying its molecular weights and monomer ratios to ameliorate its degradation rate [52].

PHAs are aliphatic polyesters synthesized through the polymerization of b-, g-, and d-hydroxyalkanoic acids obtained from the fermentation of sugars and lipids from various feedstocks [53]. PHAs are polymerized by bacteria, which can synthesize them under stressful conditions as a carbon and energy reserve [54]. Large-scale production of PHAs is expensive, requiring fermentation, isolation, and purification processes that limit their widespread use [55,56]. Nonetheless, PHAs are driving the growth of the biodegradable bioplastics market, with production capacity expected to triple in the next five years [57]. Poly-4-hydroxybutyrate (PHB) and poly(3-hydroxybutyrate-co-3-hydroxyvalerate) (PHBV) are the most commonly used PHAs: PHB has a high elastic modulus and better barrier properties than PLA. However, it is brittle and has lower thermal stability. PHBV has PP-like properties and is commercially available added with a hydroxyvalerate (HV) that is able to confer more flexibility than PHB [58]. Like PLA, PHAs find various applications in the packaging and biomedical fields as single-use items. PHB and PHBV have been investigated for their potential use as bioresorbable materials for surgical sutures, wound dressings, tissue scaffolds, bone fracture fixation plates, and porous sheets for tissue regeneration in injured soft tissues [53]. The biodegradability of PHAs depends on factors, such as chain configuration, crystallinity, and processing conditions. Another important advantage of PHAs is their high degradation rate in marine environments [59].

## 3. Routes for MPs and NPs Adsorption, Tissue Accumulation, and Biological Effects

All plastics, both biodegradable or not, can initiate their degradation process reducing the manufacture size into smaller particles known as MPs and NPs. Studies on preferential adsorbing routes, tissue internalization, accumulation, and molecular mechanisms of penetration are important to assess toxic potential and to better understand the capability of MPs and NPs to target specific tissues. Indeed, there is growing evidence of the harmful effects of MPs/NPs on organisms ranging from plants and fish to microorganisms and animals [60]; thus, MPs/NPs have been studied for their potential hazards and health implications for humans even though investigations are still in infancy.

### 3.1. Dermal Route

Due to the presence of bioplastic MPs/NPs in cosmetic products, such as body and facial scrubs, creams, soaps, and other beauty products, as well as in microfibers and drug delivery systems for dermal application, the skin represents a potential route for human exposure [61]. For instance, hair follicles, sweat glands, and injured skin represent all possible entry routes [61]. Some interesting data on conventional NPs’ dermal penetration were obtained by Campbell et al. [62], who demonstrated that PS-NPs reach a depth of approximately 2–3 µm from the top layers of pig skin tissue. Furthermore, Vogt et al. [63] demonstrated the presence of 40 nm-diameter fluorescent PS-NPs in the perifollicular tissue of human skin explants undergone cyanoacrylate follicular stripping, indicating a size-dependent absorption of transcutaneous application of particles by Langerhans cells around hair follicles. By using ex vivo human skin samples, Zou et al. [64] investigated the effects of skin condition, incubation temperature, particle size, and vehicle solutions on NPs’ uptake. They found that the use of dimethyl sulfoxide as a vehicle led to deeper penetration of PS-NPs compared to ethanol and water. Tape stripping also allowed deeper penetration, but only until the granulosum layer. Similarly, in excised human skin samples, Jatana et al. [65] observed that ingredients present in skincare lotions can facilitate bioplastic particle penetration of PCL-NPs corroborating that skin conditions and vehicles influence the uptake of these NPs by the skin. Considering animal and human skin differences in their anatomical structure, results obtained by ex vivo human samples should be considered more reliable [66]. The overall observations suggest that the dermal penetration route is not preferred; thus, bioplastic MPs/NPs are not largely used in pharmaceutical applications for transdermal drug delivery. However, the effects of this route on human health received very little attention and should be certainly further investigated. 

### 3.2. Inhalation

The fact that MPs and NPs can be transported in the air over long distances favoring their presence both in aquatic and terrestrial environments has been well documented in conventional plastics, whereas studies on bioplastics are not [67,68,69]. Due to their size, MPs/NPs can be easily inhaled and can reach the lungs, where they should be removed through the mucociliary escalator or phagocytosis by alveolar macrophages [70], but MPs/NPs can overcome these defense mechanisms and remain on the alveolar surface, causing lung damage. In vitro studies report a positive correlation between exposure to MPs and the development of pulmonary inflammation and cancer [70,71]. Exposure to MPs (4.06 ± 0.44 μm) to human lung epithelial cells (BEAS-2B) has been shown to induce the formation of reactive oxygen species (ROS), inflammatory process, and cytotoxic effects, as well as a decrease in transepithelial electrical resistance [72]. The onset of these harmful processes is affected by MPs/NPs’ properties, such as hydrophobicity, surface charge, and functionalization, that influence their absorption and clearance in the lungs. Among these properties, particle size rather than particle kind has a great relevance: the smaller the particles, the greater the effect and distribution (see Table 1). For example, in rat models, the instillation of ultrafine PS-NPs of different sizes (from 64 to 535 nm) caused lung inflammation. This effect was mediated by an excessive neutrophil influx and increased pro-inflammatory proteins and lactate dehydrogenase in bronchoalveolar lavage (BAL) [73]. Accordingly, in alveolar cell lines (A549), the treatment with PS-MPs determined an increase in IL-8 mRNA expression after 2–4 h of treatment [73]. In addition, it has been found that a mixture of MPs (MPs < 50 μm) taken from environmental plastic waste can accumulate in A549 cells exposed in vitro and induce oxidative stress, genotoxicity, and alterations in gene expression [74,75]. Similar results were obtained by exposing mice to particles from tire wear by inhalation [76]; in this case, reduced ventilatory functions and exacerbated pulmonary fibrotic injury were observed [76].

The alveolar epithelium barrier is thin enough for NPs to enter blood capillaries, allowing them to disperse throughout the human body and potentially cross the BBB [77]. Both conventional and bioplastic particles, depending on their hydrophilicity, size, and surface charge, can translocate and enter the circulation, probably more easily when endothelial and epithelial permeability is increased during immune/inflammation response. In fact, a research group aiming to develop improved mucosal immunization strategies exposed BALB/c mice to PS carboxylate MPs (1.1 µm in size) by intranasal delivery. Treated mice exhibited the presence of PS-MPs in the nasal-associated lymphoid tissues (NALTs) and draining cervical lymph nodes after 7 days. In addition, MPs’ accumulation in the spleen was also observed corroborating that the spleen can act as an inductive site following bronchopulmonary deposition [78].

Since the nasal route is also used for drug delivery to the brain [79], it can be argued that once MPs/NPs are unintentionally inhaled, the olfactory nerve might permit the transit of NPs to the CNS. Different studies have demonstrated that various nanomaterials, once in contact with the olfactory epithelium, can be transported to the brain through olfactory neurons inducing detrimental effects such as brain inflammation [80,81,82]. However, few studies have investigated this exposure route, even if some authors confirmed the presence of MPs and NPs in the respiratory system and their passage through the BBB [67,70]. Collectively, both in vitro and in vivo research suggest that MPs/NPs have toxic effects on the respiratory tract and lungs, leading to inflammation and lung fibrosis.

**Table 1:** Preferential accumulation sites of plastic or bioplastic MPs/NPs’ exposure in vivo and their biological effects.

Exposure Route	Type	Size	Accumulation	Biological Effects	Ref.
***Dermal contact***	NPs	20–200 nm	Hair follicular openings	ND	[83]
	NPs	40 nm 750 nm 1500 nm	Langerhans cells and epidermal cells	ND	[63]
***Inhalation***	NPs	64 nm	Lung epithelium	Lung inflammation, excessive neutrophil influx, proinflammatory proteins	[73]
	NPs	<1 μm	Pulmonary alveolar units	Pulmonary parenchymal lesion, alveolar stenosis, fibrous tissue hyperplasia, perivascular, lymphocyte, infiltration, ꜜ E-cadherin expression, ꜛ collagen deposition	[76]
	MPs	1.1 μm	NALTs, mediastinal lymph node, spleen, bronchopulmonary deposition	ꜛ Immunological response	[84]
***Ingestion***	MPs	10–150 μm	Colon and duodenum	Gut microbiome alterations, intestinal inflammation, ꜛ pro-inflammatory cytokines, intestinal glands disruptions	[85]
	NPs	100 nm	Stomach, small and large intestines, kidney, lungs	Liver immune cells infiltration, hepatocyte vacuolization, pulmonary interstitial fibrosis, renal tubular atrophy, ileum epithelium disruption, colon lymphocyte aggregation, neuron alterations, testicular atrophy	[86]
	MPs/NPs	50 nm 500 nm 5 μm	Intestine, liver, kidney, testis, brain	ꜛ Inflammatory factors	[87]

Abbreviations: MPs, microparticles; NPs, nanoparticles; PS, polystyrene; TW, tire wear; PE, polyethylene; NALTs, nasal-associated lymphoid tissues. ꜛ increase; ꜜ decrease.

### 3.3. Ingestion

The most significant MPs/NPs exposure route for humans is ingestion. MPs/NPs are present in food and drink containers, and in edible products, and have been found in the gastrointestinal system of fishes [5,84,88]. As in skin and lungs, in gastric cells, smaller-sized particles have more possibilities to be absorbed. Many studies are available in the current literature examining the effects of conventional plastics and their accumulation in the gastrointestinal tract, while data on the consequences of the ingestion of bioplastics are still lacking. In a study carried out by Banerjee et al. [89], the toxicity of PS MPs/NPs was examined in SNU-1 human gastric epithelial cells. The study revealed that smaller particles (50 nm) were taken up more avidly by the cells than larger particles (1000 nm), increasing cell cytotoxicity, apoptosis, and necrosis [89]. To gain a better understanding of the chronic exposure to MPs/NPs through the gastrointestinal tract, Domenech et al. (2021) [90] investigated the effects of 50 nm PS particles on CaCo-2 colon cancer cells. After 8 weeks of treatment, it was found that 20% of the cells had taken up and internalized the particles and exhibited altered expression of oxidative-stress-related genes. Conversely, another study [85] demonstrated that 5 weeks of exposure of mice to PE-MPs by ingestion led to gut microbiome alteration and intestinal inflammation. The MPs/NPs-induced effects were not limited to the gastrointestinal tract since the potential ability of NPs to permeate the gut epithelium and pass into the systemic circulation can lead to both damage and disruption of the intestinal barrier and accumulation in other tissues and organs far from the primary route of exposure [91]. Accordingly, Xu et al. [86] investigated the uptake mechanism of NPs in mice demonstrating that PS-NPs’ absorption occurs through clathrin-mediated endocytosis. Furthermore, a reduction in occludin and zonula occludens-1 (ZO-1) was observed in Caco-2 cells, thus suggesting the potential ability of these particles to disrupt the intestinal barrier. By passing the intestinal epithelium, particles could enter the circulation accumulating in the spleen, liver, lung, and brain [86]. Han et al. [87] demonstrated that PS particles combined with di-(2-ethylhexyl) phthalate (DEHP) accumulated in mouse organs, such as intestines, livers, kidneys, testis, and brain. The accumulation of NPs in the brain after ingestion probably takes place through the lymphatic or blood circulation, the translocation through the BBB—which appears to be affected by enhanced permeability induced by PS-MPs—and the transport up to the brain parenchyma [21,92,93].

Noteworthy, after entering the circulatory system, MPs and/or NPs could also affect blood and immune cells. Indeed, we have shown that PLA microbeads (size 1 ± 0.2 μm) used as drug delivery systems can be internalized by human monocytes with an efficiency of 30%; but after PLA-MPs phagocytosis, cell apoptosis increased in a dose-dependent manner [94]. Instead, empty microcarriers of PLGA (mean size 827 ± 68 μm) at a concentration of 12.5 mg/mL did not induce any cytotoxic effect on human peripheral mononuclear cells (hPBMCs) from healthy donors [95]. By reducing the size of PLA- and PLGA-NPs (0.4–3 μm), both PLA and PLGA NPs exhibited low cytotoxicity in Chinese hamster ovary (CHO) cells and in hPBMCs, corroborating, as in conventional NPs, that the particle size is fundamental to cause biological effects. However, at the same concentration, PLGA affected cell viability more than PLA [96]. 

**Figure 2:**
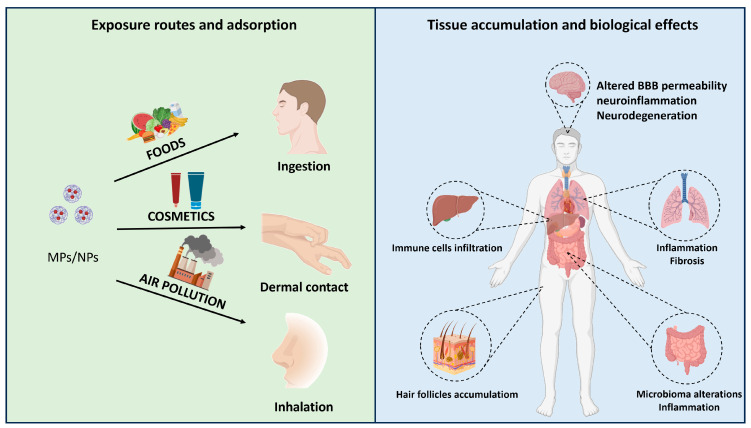
Overview of microplastics (MPs) and nanoplastics (NPs) destiny from the environment up to biological effects. BBB, blood–brain barrier.

Studies on contamination routes, deposition, and related health risks of exposure to bioplastic MPs/NPs are widely unexplored. Few data indicated that gut enzymatic hydrolysis of PLA-MPs in mice generated smaller particles by competing for triglyceride-degrading lipase. PLA oligomers and their NPs accumulate in the liver, intestine, and brain, inducing intestinal damage and acute inflammation [97]. Details regarding the size and distribution of MPs/NPs in tissues are given in Table 1, while a schematic representation showing different aspects of adsorption, tissue accumulation, and biological effects is summarized in Figure 2.

## 4. Bioplastic-Based Polymeric NPs for Drug Delivery in the Brain

### 4.1. Bioplastic MPs/NPs as Drug Delivery Systems

Significant advancements in nanomedicine have led to the development of drug delivery systems based on bioplastic MPs/NPs. These systems offer several advantages, including increased drug shelf-life, targeting to the specific organ/tissue, reduced therapeutic dose administrations, improving drug effectiveness, and minimizing side effects. Among drug delivery systems, the PLA and PLGA-NPs are the most extensively studied [98], because of their high drug-loading capacity, good biocompatibility, biodegradability, and tunable release properties. PLA/PLGA pharmaceutical formulations were developed for sustained delivery of several drugs used in cancer treatment, anti-inflammatory compounds, and drugs for neurological disease [99,100], while many others are still in phase II or III of clinical trials [101] (see Table 2). 

In the field of treatment of neurodegenerative diseases, pharmaceutical formulations have been often unsuccessful due to the BBB that is very difficult to cross [102]. Instead, PLA/PLGA NPs have emerged as a promising solution for delivering drugs to the brain, as they can overcome the physiological barrier through different strategies, such as transcytosis pathways (Trojan horse strategy) or by escaping the efflux pumps by bearing specific ligands onto the particle surface [10]. In general, the smaller size coupled with surface modifications improved formulation pharmacokinetics, enhancing cell uptake and, consequently, drug absorption [103,104].

**Table 2:** Bioplastic MPs/NPs-based drug delivery systems approved by the FDA for disease treatments.

Name	Bioplastic	Loaded Drug	Therapeutic Application	Company	FDA Approval (Date)	Ref.
Lupron Depot^®^	PLGA	Leuprolide acetate	Prostate cancer, endometriosis	Takeda–Abbott Products (Osaka, Japan)	1989	[105]
Atridox^®^	PLA	Doxycycline hyclate	Chronic adult periodontitis	Tolmar (Fort Collins, CO, USA)	1998	[99]
Sandostatin Lar^®^	PLGA	Octreotide acetate	Acromegaly	Novartis (Mulgrave, VIC, Australia)	1998	[99]
Trelstar^®^	PLGA	Triptoreline pamoate	Advanced prostate cancer	Allergan (Gordon, NSW, Australia)	2001	[99]
Risperdal Consta^®^	PLGA	Risperidone	Schizophrenia, bipolar I disorder	Janssen (Beerse, Belgium)	2003	[106]
Vivitrol^®^	PLGA	Naltrexone	Alcohol dependence	Alkermes (Waltham, MA, USA)	2006	[100]
Signifor Lar^®^	PLGA	Pasireotide pamoate	Acromegaly	Novartis (Mulgrave, VIC, Australia)	2014	[99]
Sublocade^®^	PLGA	Buprenorphine	Opioid disorder	Indivior (Richmond, VA, USA)	2017	[99]
Triptodur Kit^®^	PLGA	Triptorelin pamoate	Central precocious puberty	Arbor (Mulgrave, VIC, Australia)	2017	[99]
Scenesse^®^	PLGA	Afamelanotide	Prevention of phototoxicity in erythropoietic protoporphyria	Clinuvel (Melbourne, VIC, Australia)	2019	[107]
Durysta^®^	PLA/PLGA	Bimatoprost	Glaucoma, open-angle, intraocular hypertension	Allergan (Gordon, NSW, Australia)	2020	[107]

Abbreviations: PLGA, polylactic co-glycolic acid; PLA, polylactic acid; PCL, polycaprolactone.

### 4.2. Techniques for Fabricating Bioplastic MPs/NPs

**Figure 3:**
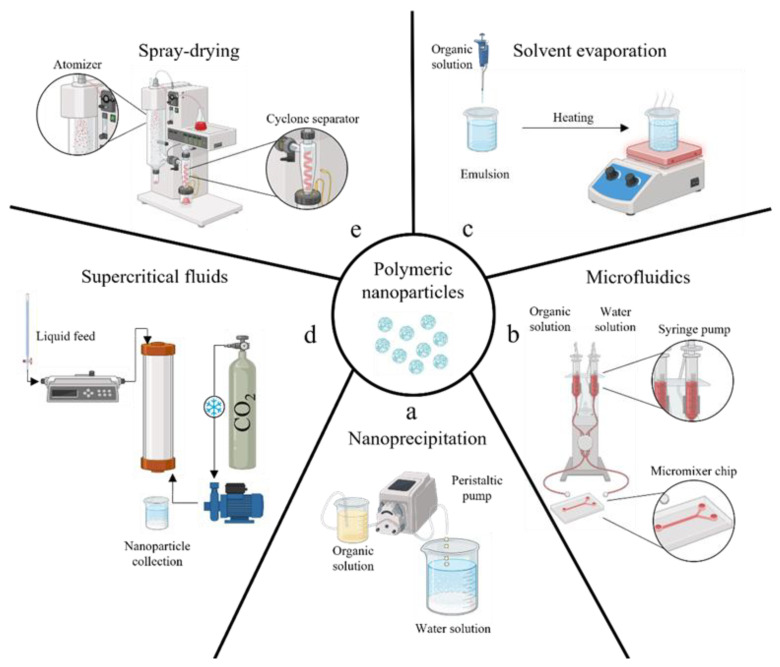
A schematic illustration of the principal manufacturing methods to produce polymeric micro/nano-particles: conventional nanoprecipitation by anti-solvent effect (**a**), nanoprecipitation enhanced by adopting microfluidics channel (**b**), solvent extraction/evaporation of emulsion (**c**), supercritical fluids emulsion extraction (**d**), spray drying (**e**).

**Figure 4:**
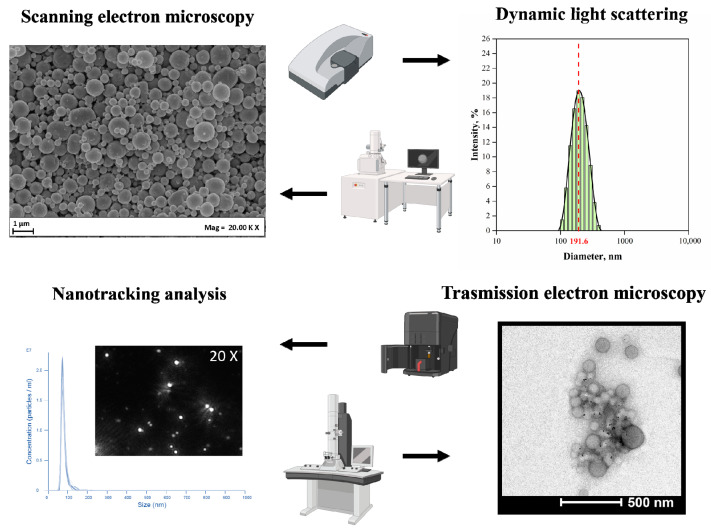
Chemical and physical characterization of the polymeric nanoparticles.

Several technologies have been described for the fabrication of bioplastic MPs/NPs, such as nanoprecipitation, solvent evaporation or extraction, spray drying, and supercritical fluids [108,109]. All described technologies are schematically illustrated in Figure 3, and examples of MPs/NPs’ characterizations by laser scattering (size distribution curve) or SEM/TEM (micrographs) are shown in Figure 4.

The nanoprecipitation process involves the precipitation of a polymer from a water-miscible organic solvent by mixing it with an aqueous medium, acting as an anti-solvent (see Figure 3). The size can be controlled by adjusting different parameters, such as organic solvent, polymer concentration, and surfactant amount in the water [110,111,112]. Since proper mixing is required between the two fluids, microfluidic systems have been recently described as promising tools for this task [113]. Indeed, the nanoprecipitation within micrometering channels assures strict control over the particle size by varying pump flow rates and micromixer geometry [114,115]; however, the method is unsuitable for encapsulating hydrophilic drugs into the NPs [112] (Figure 3b).

Solvent evaporation from emulsion is also widely used; that is, when emulsions undergo evaporation or extraction, the dispersed oily droplets within the surrounding water phase can solidify due to organic solvent removal, developing micro/nanocarriers (Figure 3c) [116,117,118]. Physical properties of resulting particles can be modulated by varying surfactant type and concentration, stirring rate, and solvent evaporation conditions; the method can encapsulate hydrophobic and hydrophilic drugs depending on the use of single or multiple emulsions; however, the evaporation step demonstrates fluctuations in reproducibility from one batch to another [119,120]. At the same time, extraction requires comparatively large amounts of a second solvent, with the related issue of further solvent recovery. Both processes require processing times of several hours that can promote aggregation phenomena between the droplets, producing carriers with a larger polydispersity [121]. It should also be noted that, despite the widespread use of solvent evaporation/extraction processes to prepare polymeric carriers, there are no established standard protocols, and each preparation follows its own set of procedures. Finally, this may not be suitable for temperature-sensitive drugs due to the risk of degradation during the solvent evaporation step as well as poor encapsulation efficiency of high water-soluble compounds reported [95].

Dense carbon dioxide technologies have been proposed to produce bioplastic MPs/NPs for different drug delivery purposes [122,123,124,125] or tissue engineering [96,126]. Supercritical emulsion extraction (SEE) technology operating in a continuous layout using a counter-current packed tower [127,128] was described for both PLA and PLGA carriers’ fabrication. In detail, an emulsion that contains polymer undergoes solvent removal using supercritical carbon dioxide as an extraction fluid (Figure 3d). The dense-gas extraction technology ensured improved performances, such as a better batch-to-batch reproducibility [129], more accurate carrier size control—thanks to a fixed droplet shrinkage without aggregation phenomena [130], lower solvent residue, and better-controlled encapsulation efficiency [95,96,131].

Nano spray drying is a relatively recent technique enabling the single-step fabrication of bioplastic MPs/NPs starting from a small sample volume. In this process, a liquid solution containing both the polymer and the drug is transformed into tiny droplets through atomization by a spray nozzle. Subsequently, these droplets undergo rapid drying as they are exposed to a stream of hot air or inert gas. As a result, solid particles are formed through the evaporation of the solvent (Figure 3e). In this case, the particle size and especially the size distribution may be impacted by electrospray process parameters, such as nozzle diameter, spraying rate, and drying temperature [132]. This method can yield nanoparticles with a narrow size distribution and high drug-loading capacity [133]. In contrast, conventional spray drying can produce carriers with lower encapsulation efficiency and larger size and granulometry.

To sum up, the choice of suitable technology for the production of bioplastic MPs/NPs for drug delivery will depend on several factors, including polymer or drug solubility, desired carrier size, distribution, and shape or surface charge.

### 4.3. Bioplastic MPs/NPs Delivery to the Brain 

In general, a drug delivery system facilitates the attainment of the desired therapeutic response of an active substance by enhancing its bioavailability at the target site while ensuring optimal effectiveness and safety [134]. Due to the presence of the BBB, which limits the entrance of external substances into the brain, many efforts are being made to use bioplastic NPs as drug carriers to the brain. Although parenteral administration is the prevalent route for bioplastic MPs/NPs, its effectiveness for brain drug delivery is still in development. Intranasal administration is an alternative route to bypass the BBB as it allows direct access to the brain, even though its clinical application is hindered by the limited knowledge of nanoparticle deposition and absorption in this anatomical site [135]. Intracranial administration, by bioplastic MPs/NPs’ injection directly into the brain tissue or cerebrospinal fluid, is a direct and effective route for brain drug delivery using bioplastic MPs/NPs. However, this route is invasive and may cause tissue damage or inflammation [136]. Intrathecal administration is another direct route for brain drug delivery that involves the injection of bioplastic MPs/NPs into the spinal cord or cerebrospinal fluid [137]. This route accounts for the targeted delivery of NPs to the brain and has shown to be the most promising for brain tumors and neurodegenerative disease treatment [137]. Overall, the intravenous route remains the preferred choice as those mentioned before are too invasive. As a result, several strategies have been devised to overcome the BBB and improve the delivery of bioplastic MPs/NPs to the brain. One strategy is to modify the surface of bioplastic MPs/NPs with BBB-penetrating molecules, such as peptides, antibodies, or aptamers. These modifications can increase the affinity of NPs to the BBB and enhance their transport across the barrier [138]. Another strategy involves employing ultrasound or magnetic fields to increase BBB permeability and enhance the transport of bioplastic MPs/NPs’ carriers [139].

Recently bioplastic MPs/NPs have been engineered to overcome the BBB or target specific cell types in the brain, such as neurons or glial cells. These targeted nanoparticles can enhance drug delivery to specific regions of the brain and reduce off-target effects [140]. Essentially, there are three distinct strategies to accomplish this objective: adsorptive-mediated transcytosis, transporter-mediated transcytosis, and receptor-mediated transcytosis. The first mechanism can be facilitated through electrostatic interactions between the negatively charged components present on the luminal surface of cerebral endothelial cells and the cationic groups or conjugating specific compounds, such as lectins, cardiolipin, and heparin to the NPs surface [141]. Another approach for delivering drugs to the brain is utilizing transporter-mediated transcytosis. Indeed, it is feasible to synthesize nanoparticles with surface-conjugated molecules (glucose and its analogs, glutathione, and amino acids) that exhibit strong recognition by the transporters that are overexpressed in brain endothelial cells [142]. The last approach to access brain tissue involves the use of receptors overexpressed within the BBB. In more detail, transferrin, lactoferrin, low-density lipoproteins, and nicotinic acetylcholine receptors are commonly employed receptors to achieve receptor-mediated transcytosis across the BBB [140].

Finally, the use of prodrug strategies can also enhance the delivery of bioplastic MPs/NPs to the brain. Prodrugs are inactive precursors of active drugs that can be activated within the brain by specific enzymes. In fact, hydrophilic and high-molecular-weight compounds cannot traverse the BBB or the blood–cerebrospinal fluid barrier (BCSFB) through paracellular pathways. In contrast, lipophilic solutes can passively permeate these barriers [143]; hence, hydrophilic drugs can be chemically modified into lipophilic prodrugs by concealing polar functional groups. This approach can increase the brain concentration of active drugs while reducing their systemic toxicity [144]. 

Altogether, the described strategies offer promising solutions to overcome the BBB and enhance the delivery of bioplastic NPs to the brain.

## 5. Biological Effects of Bioplastic NPs Loaded Carriers in the Brain

### 5.1. Bioplastic MPs/NPs for Brain Diseases: Advantages

Several PLGA-NPs have been developed for Alzheimer’s disease (AD) treatment. Yusuf et al. [145] designed PLGA-NPs loaded with the phytochemical compound thymoquinone (TQ). The TQ-loaded PLGA-NPs, coated with polysorbate 80, successfully crossed the BBB and significantly increased superoxide dismutase (SOD) activity in male albino mice [145,146]. Furthermore, Xu et al. [147] synthesized Tween 80-coated methoxy poly (ethylene glycol) PLGA-NPs loaded with rhynchophylline (RIN), a spirocyclic alkaloid with neuroprotective effects, to target the brain for AD treatment. In vitro and in vivo studies demonstrated that both these bioplastic NPs had a high transport across BBB and improved survival rate of neuronal cells [148]. Since AD is linked to Aβ aggregation, PEGylated bioplastic NPs loaded with an Aβ1–42 antibody have been developed and its efficacy has been investigated in a transgenic AD mouse model [149]. The treatment led to a significant improvement in memory and to a reduction in Aβ levels in the brain, indicating the potential of this approach for treating AD.

Even for the treatment of Parkinson’s disease (PD), several PLGA-NP systems have been developed. This neurodegenerative disorder is characterized by tremors, dyskinesia, and motor impairments. The appearance of these symptoms is attributable to a degeneration of dopaminergic neurons within the *substantia nigra* with consequent destruction of the nigrostriatal pathway [150,151,152]. Current therapies for PD management aim to enhance dopamine levels; in fact, the gold standard for the treatment of PD is the administration of L-Dopa, a dopamine precursor [153]. In line with such strategies, Monge-Fuentes et al. [154] developed bioplastic NPs loaded with dopamine and composed of albumin and PLGA. This nano-system, thanks to albumin, can cross the BBB better than free L-DOPA. PD mice, administered with these L-DOPA-loaded bioplastic NPs, show augmented levels of dopamine and improvements in motor symptoms [154]. Accordingly, PLGA-NPs, conjugated with wheat germ agglutinin (that enhances absorption via the nasal cavity) and loaded with L-Dopa, showed a significant improvement in the drug delivery to the brain together with better therapeutic efficacy and lower side effects [155].

More complex bioplastic NP systems have been developed consisting of multilayer hybrid PLCL-NPs encapsulating together L-Dopa, Tenoxicam as an anti-inflammatory drug, and Lamotrigine as a neuroprotective agent. This formulation was able to act on more than one complication associated with PD and improved cognitive abilities in PD-induced rats [156].

Few PLGA-based drug delivery systems have been developed for the treatment of other neurological disorders, such as Huntington’s disease (HD), amyotrophic lateral sclerosis (ALS), and multiple sclerosis (MS). HD is a genetic disease characterized by an accumulation of the mutant huntingtin (mHTT) protein in nerve cells [157]. Symptoms consist of motor impairment, psychiatric disorders, and behavioral disorders as a consequence of the progressive loss of nerve cells and brain mass; the neurons of the striatal part of the basal ganglia are the most affected [157]. Currently, there are no approved therapies to delay the onset and progression of HD. Therapeutic approaches targeting the cause of HD, i.e., the CAG-expanded *HTT* gene and its products, or downstream processes associated with the pathogenesis of HD, are still in the clinical development [158]. Joshi et al. [159] designed PLGA-NPs coated with polysorbate 80 and loaded with oligonucleotides able to inhibit the *HTT* gene. These authors, using a Drosophila model of HD, reported improvements in motor performance without signs of toxicity [159,160].

For the treatment of ALS, a neuromuscular disease in which motor neuron loss occurs due to an altered expression of retinoid signaling [161,162] realized adapalene-loaded PLGA-NPs. These bioplastic NPs, tested in OD1G93A transgenic mice, showed an activation of the retinoid signaling pathway in the CNS with an improvement in neuroprotection and motor performance. 

MS is known as a disease in which the immune system attacks the myelin sheath, an important component of axonal membranes, inducing progressive loss of neuronal structure and functions [163]. In this regard, recently Gholamzad et al. developed PLGA-NPs conjugated with myelin oligodendrocyte glycoprotein (PLGA-MOG) [164]. These particles were then intravenously administered to a C57BL6 mouse model of MS. PLGA-MOG NPs were able to ameliorate clinical symptoms and autoimmune responses, reducing the infiltration of immune cells within the brain.

Bioplastic NPs have emerged also as a strategy for the treatment of brain cancers such as glioblastoma which is considered the most aggressive form of CNS tumors [165,166]. In a study carried out by Maksimenko et al. [167], it emerged that PLGA-NPs (size 110 nm), loaded with doxorubicin and coated with poloxamer 188 (Dox-PLGA), crossed the BBB more efficiently than doxorubicin alone in a xenograft rat model. Dox-PLGA-NPs were able to significantly reduce intracranial 101.8 glioblastoma size after 2 weeks of treatment with 3 × 1.5 mg/kg bw (as doxorubicin) after tumor implantation. Accordingly, some other studies carried out to improve the transport of anti-proliferative drugs across the BBB have shown that transferrin-conjugated magnetic silica PLGA-NPs loaded with doxorubicin and paclitaxel stimulate ROS and TNF-α production inducing time and dose-dependent cytotoxicity against glioma cells in vitro and in vivo [168]. The effectiveness of conjugated nanocarriers loaded with chemotherapeutic drugs has also been demonstrated for PLGA-NPs—loaded with morusin (PLGA–MOR) and conjugated with chlorotoxin (CTX), a peptide that binds specific chloride channels and matrix metalloproteinase (MMP-2) [169]. In this case, the treatment of U87 and GI-1 glioma cells with PLGA–MOR–CTX NPs resulted in enhanced anti-proliferative effects by inhibiting MMP activity and inducing cytoskeletal alterations, ROS generation, and apoptosis [169].

The overall data indicate undoubtedly that bioplastic NPs represent a promising strategy for the treatment of neurodegenerative disorders and brain tumors, since they can cross or bypass the BBB delivering drugs in a specific manner and preserving their efficacy. Despite the numerous efforts and encouraging results obtained until now, we are still far from the effective use of these formulations, as no bioplastic NPs-based system is currently on the market [170]. The only available data concern preclinical studies (Table 3), and many aspects mainly linked to pharmacokinetics (ADME) still need to be clarified [171,172].

### 5.2. Bioplastic MPs/NPs for Brain Diseases: Drawbacks

Despite bioplastic MPs/NPs seeming effective as drug delivery systems for the brain, their toxicity is largely unknown, and available information is strictly related to conventional NPs from industrial production. In this regard, several studies have demonstrated that NPs could be involved in neuroinflammation and increase oxidative stress [172,174,175]. The generation of ROS could cause damage to lipids, nucleic acids, proteins, and other essential biomolecules, leading to mitochondrial dysfunction and cell death by altering signaling pathways [176]. Bioplastic MPs/NPs’ exposure is also responsible for activation of microglia cells and astrocytosis, leading to neuroinflammation and neuron function impairments [177]. However, to date, toxicological data are still few and limited to cautious hypotheses referring to systemic toxicity. In addition, the only available data differ from each other by the administration route, dose metrics, particle size, and loaded drug [178], making it difficult to draw general conclusions. Indeed, despite PLGA and PLA biocompatibility and biodegradability, bioplastic MPs/NPs for drug delivery, when produced on a nanoscale dimension, could have toxic effects [179] that are far from being fully understood. Bioplastic MPs/NPs may have dimensions quite far from cells (1:10; 1:100), and this implies that they could easily interfere with the activity and metabolism of neurons, microglia, and astrocytes [180,181]. A study focused on the in vivo neurotoxicity of polysorbate 80-chitosan NPs, after intravenous injection in rats, showed an accumulation in several brain areas such as the frontal cortex and cerebellum inducing neuronal death, inflammation, and oxidative stress [182]. Furthermore, the removal of bioplastic MPs/NPs by the blood can occur very slowly, thus promoting their accumulation with potential side effects [183]. From this point of view, it is crucial to analyze damaging effects at different levels, including the ultrastructure of the cell, since this level represents the boundary between molecular and spatially organized levels of the body. NPs interact with the cell precisely at this level, and dysfunction of cells or the action of redundant and regulatory systems could play a negative role in smoothing out the effectiveness of treatments. Another important factor is the toxicity related to degradation. PLA or PLGA degradation products, such as lactic or glycolic acid, could lead to tissue acidification when accumulated. Furthermore, PLGA has been reported to be much more responsible for inflammation because of its faster degradations (weeks) with respect to PLA (months) [184]. Increased levels of lactate could interfere with bioenergetic processes such as glycolysis and oxidative phosphorylation, and it is known that brain alterations in energy metabolism are associated with schizophrenia and bipolar disorders [185]. Furthermore, bioplastic MPs/NPs once in contact with biological systems could interact with neighbor biomolecules. As a result, these interactions could lead to the formation of the so-called “protein corona”. Protein corona affects NPs’ properties and alters their pharmacokinetics with possible aberrating biodistribution, toxicity, and mistargeting [186,187,188]. 

**Figure 5:**
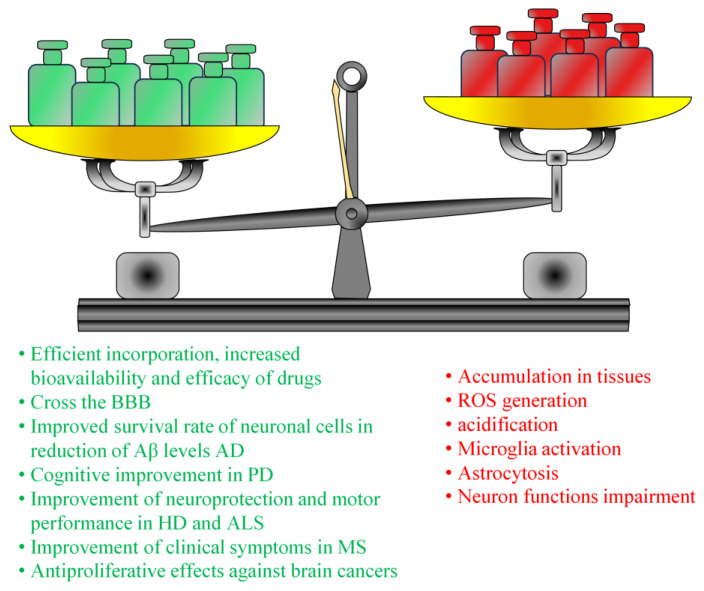
Pros and cons of bioplastic MPs/NPs drug delivery systems for brain disease management.

Other several factors could influence the neurotoxicity of bioplastic MPs/NPs. First of all, the size of polymeric NPs: all NPs with smaller sizes can penetrate the BBB more easily and have a higher surface area which can increase their interactions with brain cells and cause toxic effects [189]. The surface chemistry of bioplastic MPs/NPs is another factor that can influence their neurotoxicity. Surface modifications such as the addition of targeting ligands or PEGylation could alter the surface charge and hydrophobicity of NPs, affecting their cellular uptake but also their ability to interfere with endogenous cell signaling pathways [140]. Furthermore, intravenous injection can cause the accumulation of bioplastic MPs/NPs in the liver, spleen, and other organs, leading to potential systemic adverse effects. Finally, higher doses and more frequent administrations can increase the negative health effects, while lower doses and infrequent administration can reduce the toxic risk [190]. In summary, bioplastic MPs/NPs possess good properties for successful use in pharmaceutical preparations for the treatment of brain diseases; but it is important to understand the balance between the therapeutic and toxic actions of these drug carriers, because their potential clinical use still represents a matter of concern (Figure 5).

## 6. Conclusions

In the light of environmentally friendly solutions, nowadays bioplastics are considered a valid alternative. However, their faster biodegradability to smaller particles (MPs and NPs) poses a potential health risk for animals and humans since they can accumulate in tissues and organs altering homeostasis and physiological functions. Furthermore, bioplastic MPs/NPs are well-studied and investigated as carriers for drug delivery and can easily pass through physiological barriers because they have been developed for these purposes, such as drug loading to assure its proper delivery and improve its bioavailability at the target site. 

Despite these bioplastic MPs/NPs exhibit a capacity of complete biodegradation in the body and represent a useful tool for personalized and precision medicine, there are still some issues that should be addressed because side and toxic effects have not been sufficiently investigated, and the majority of data refer to the drug and not to the carriers. Knowing the exact mechanism of distribution, metabolism, and excretion is fundamental to understanding the potential toxic effect of bioplastic MPs/NPs. Until now, there exists a knowledge gap regarding this aspect, and the studies concerning their use for therapeutic purposes are far greater than those aimed at determining their toxicological profile. However, the field of nanotoxicology aimed to understand the interaction between biomaterials and living cells is growing, but studies are still few and methods and results are frequently controversial.

Taken together, all of this evidence emphasizes the importance of the risk assessment for bioplastic MPs/NPs, but it is also necessary to standardize the evaluation method to obtain a reliable validation of their toxicity. Indeed, until now, studies have been conducted using different in vivo and in vitro models, and there are no results about the effects of long-term exposure to these particles. Additionally, because the fabrication of bioplastic MPs/NPs as a delivery system differs based on the method of preparation, there is no general data available about their bio-interaction and biodistribution.

## Figures and Tables

**Table 3:** Preclinical studies on bioplastic MPs/NPs used as drug delivery systems in brain diseases.

Biopolymer	Technology	Mean Size (nm)	Drug Loaded	In Vitro/In Vivo Model	Data	Brain Disease	Ref.
Chi-PLGA	NP	136 ± 30	Lutein	-Co-culture model of BBB -Nasal mucosa -Male Wistar rats	Oxidative stress reduction	AD	[173]
PLGA	SE	226.2 ± 40	Thymoquinone	-Male rats	ꜛ SOD activity ꜜ Oxidative stress	AD	[145]
PEG-PLGA	NP	145.2 ± 43	Rhynchophylline	-bEnd-3 cells -C57BL/6 mice	-Pass through BBB -Regulate neuronal activity	AD	[147]
PEG	NP	125 ± 65	Functionalized Ab anti Aβ	-AD transgenic mice -male Tg2576	-Correction of memory deficit -Reduction in Aβ levels	AD	[149]
PLGA	SE	497 ± 353	Dopamine	-6-OHDA PD Swiss mice	-Replenishment dopamine level -Motor symptoms improvement	PD	[154]
PLGA	SE	720 ± 87	Levodopa	-PC-12 neuronal like cells -CD57/BL6 mice	-Improvement drug delivery ꜛ Therapeutic efficacy	PD	[155]
PEG-lipid-PLCL-NP	NP	150 ± 50	Dopamine/Tenoxicam Lamotrigine	-Human cortical neuronal cells-2 (HCN2) -BBB hCMEC/D3 cell line -Male Wistar rats	Cognitive improvement	PD	[156]
PEG-PLA-	SE	106 ± 5.4	Adapalene	-OD1G93A transgenic mice	-Improvement of adapalene encapsulation -Activation of retinoid signaling pathway -Improvement motor performance	ALS	[162]
PLGA	SE	521 ± 289	with MOG	-C57BL/6 mice	Ameliorates autoimmune response	MS	[164]
PLGA	SE	~110	Doxorubicina	-Male Wistar rats	Antitumor effects	Glioblastoma	[167]
PLGA	SE	250 ± 180	Chlorotoxin-conjugated morusin	-U87 and GI-1 human glioma cells	Antitumor effects	Glioblastoma	[169]

Abbreviations: PLGA, polylactic co-glycolic acid; Chi, chitosan coated; PEG, polyethylene glycol; PLCL, poly (l-lactide-co-ε-caprolactone); NP, nanoparticle; PNP, polymeric nanoparticle; MOG, myelin oligodendrocyte glycoprotein; BBB, blood–brain barrier; SOD, superoxide dismutase; AD, Alzheimer’s disease; PD, Parkinson’s disease; ALS, amyotrophic lateral sclerosis; MS, multiple sclerosis; NP, nanoprecipitation; SE, solvent evaporation. ꜛ increase; ꜜdecrease.

## Data Availability

Not applicable.
